# Supplementary material for: Network diffusion model predicts neurodegeneration in limb-onset Amyotrophic Lateral Sclerosis
Source: PLoS One. 2022 Aug 11;17(8):e0272736. doi: 10.1371/journal.pone.0272736 (PMC9371353; doi:10.1371/journal.pone.0272736)
Supplement: S1 Table — Uncorrected p-values are reported as NDM modelling uses effect-sizes (t-values) to measure the strength of atrophy. (DOCX) [file pone.0272736.s001.docx]

**S1 Table**. Cross-sectional and longitudinal regional atrophy (t-statistics) measured in ALS. Uncorrected p-values are reported as NDM modelling uses effect-sizes (t-values) to measure the strength of atrophy.

| ***ALS at baseline vs Controls*** | | |
| --- | --- | --- |
| **Brain regions** | **Measured atrophy (t-values)** | **p-values** |
| Right pars triangularis | 2.64 | 0.01 |
| Right superior frontal | 2.19 | 0.04 |
| Left lateral orbitofrontal | 2.12 | 0.05 |
| Left lateral occipital | 2.08 | 0.05 |
| Left pars orbitalis | 1.88 | 0.07 |
| ***ALS at baseline vs ALS at six-month follow-up*** | | |
| **Brain regions** | **Measured atrophy (t-values)** | **p-values** |
| Left lateral occipital | 3.56 | 0.005 |
| Right inferior temporal | 3.09 | 0.01 |
| Left inferior temporal | 2.97 | 0.01 |
| Right middle temporal | 2.93 | 0.01 |
| Left fusiform | 2.88 | 0.02 |
| Left middle temporal | 2.57 | 0.03 |
| Left thalamus proper | 2.49 | 0.03 |
| Left para hippocampal | 2.38 | 0.04 |
| Left isthmus cingulate | 2.37 | 0.04 |
| Left superior temporal | 2.33 | 0.04 |
| Right hippocampus | 2.22 | 0.05 |
| Right medial orbitofrontal | 2.07 | 0.06 |
| ***ALS at six-month vs ALS at twelve-month follow-up*** | | |
| **Brain regions** | **Measured atrophy (t-values)** | **p-values** |
| Right bankssts (cortical areas around superior temporal sulcus) | 3.53 | 0.008 |
| Left pars opercularis | 2.89 | 0.02 |
| Left putamen | 2.86 | 0.02 |
| Right precentral | 2.39 | 0.04 |
| Left caudal anterior cingulate | 2.34 | 0.05 |
| Right caudal middle frontal | 2.24 | 0.06 |
| Right caudal anterior cingulate | 2.17 | 0.06 |
