# Supplementary material for: Network diffusion model predicts neurodegeneration in limb-onset Amyotrophic Lateral Sclerosis
Source: PLoS One. 2022 Aug 11;17(8):e0272736. doi: 10.1371/journal.pone.0272736 (PMC9371353; doi:10.1371/journal.pone.0272736)
Supplement: S1 Fig — Histogram of maximum correlation between predicted and measured atrophy (at baseline A, at six-month follow-up B, at twelve-month follow-up C) in 1000 random connectomes with preserved weight, degree and strength distributions of healthy connectome. (DOCX) [file pone.0272736.s002.docx]

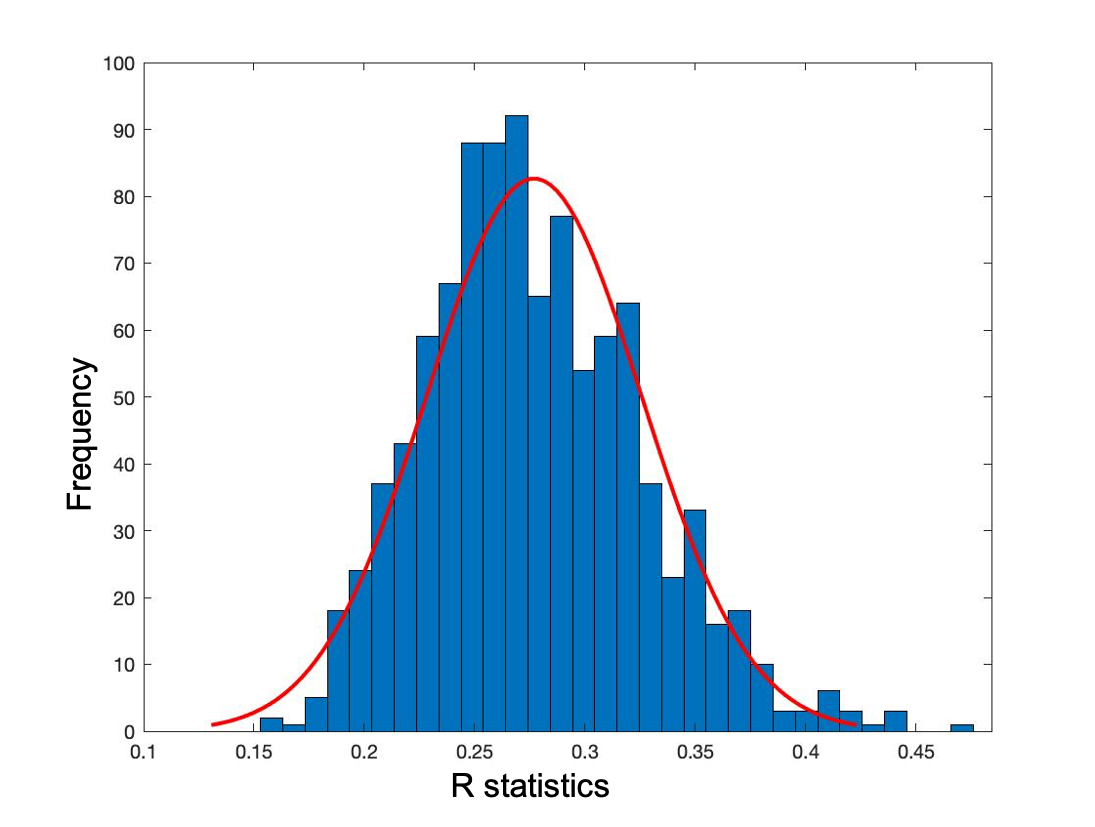


r=0.41

The correlation achieved

in healthy connectome

**A**

**B**


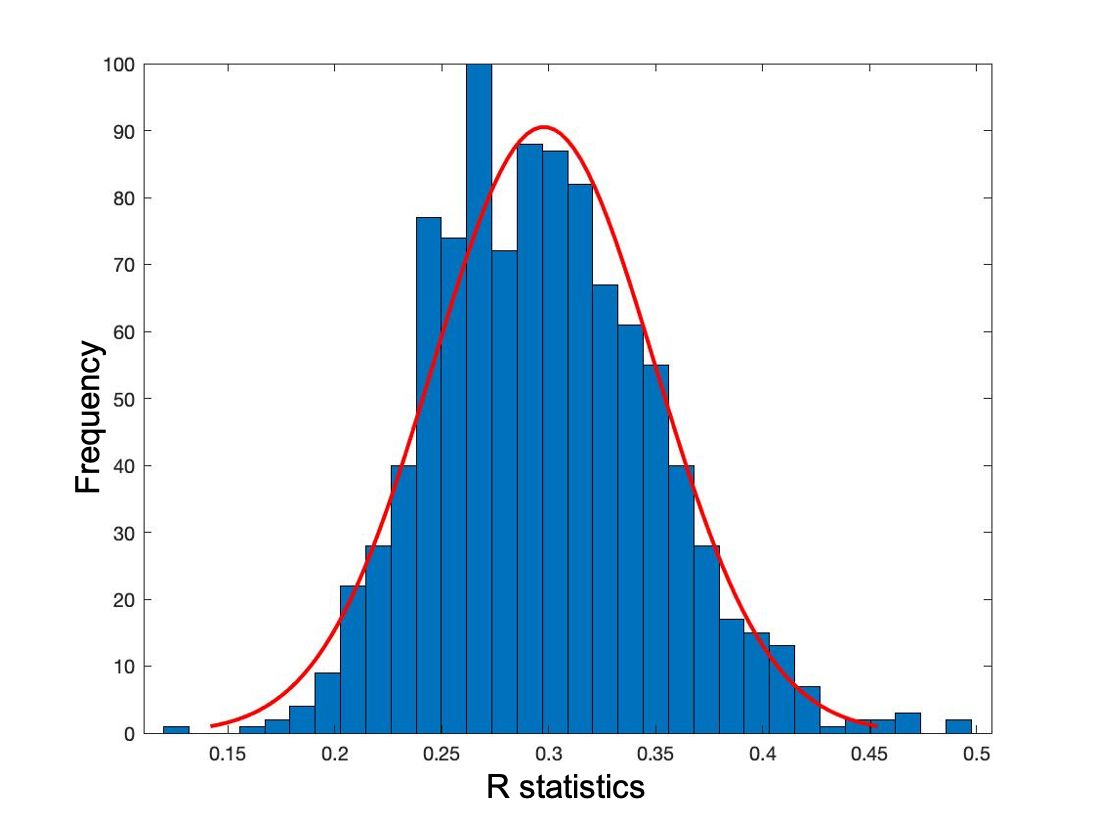


r=0.57

The correlation achieved

in healthy connectome


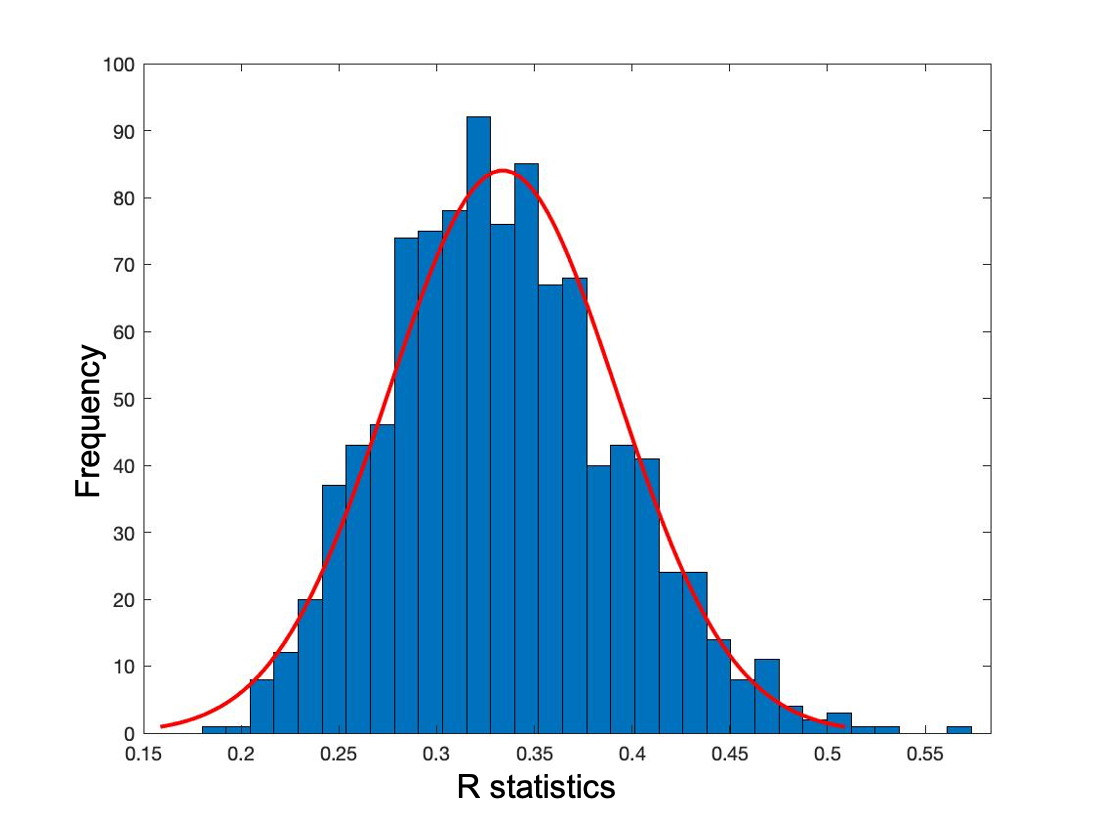


r=0.58

The correlation achieved

in healthy connectome

**C**

**S1 Fig.** Distribution of r-statistics of the relationship between measured atrophy and predicted atrophy obtained from null networks. Histogram of maximum correlation between predicted and measured atrophy (at baseline A, at six-month follow-up B, at twelve-month follow-up C) in 1000 random connectomes with preserved weight, degree and strength distributions of healthy connectome.
